# Comparing Health Care Financing in Indonesia and Thailand from 1995-2010: What Lessons Could Be Learned?

**Published:** 2013-12-18

**Authors:** Darius Erlangga, Lizheng Shi

**Affiliations:** 1 Department of Global Health Systems and Development Tulane University School of Public Health and Tropical Medicine

**Keywords:** health systems, thailand, indonesia, comparative study, health care reform, health care financing

## Abstract

**Purpose:** In 2010, the World Health Organization (WHO) released its report about health system financing and identified universal coverage as the best way to attain the right of every human being to enjoy “the highest attainable standard of health”. Over the past decade, Thailand has successfully implemented a universal health coverage scheme for its population, while its neighbor country, Indonesia, is still struggling to achieve the same goal. The purpose of this paper is to compare the health financing systems between Thailand and Indonesia. Both countries almost have similar socioeconomic conditions and suffered from severe financial crisis during the late 1990s. The objective of this study is to examine health systems in each country and to determine lessons on how health care financing can affect the health status of a population.

**Methods:** The study is based on statistical data from various publicly available resources. For analysis, the authors followed The Health Systems Assessment Approach: A How-To Manual Version 1.0 issued by Health Systems 20/20 supported by United States Agency for International Development (USAID). The countries were compared using three groups of indicators in health systems performance and functioning: 1. Health Insurance System, 2. Amount and Sources of Financial Resources, and 3. Health Outcomes and Health Workforce Density.

**Results:** In comparing the health financing of the two countries, we found that Thailand initiated much earlier health systems reforms in order to achieve universal health coverage. Indonesia, while on the right track, has moved at a slower pace than Thailand. Thailand and Indonesia have shown improving trends over time in all indicators, but Thailand outperformed Indonesia, especially in the groups of indicators regarding the amount and sources of financial resources.

**Conclusions:** One important lesson identified in this study is that health care reform is unlikely to succeed without strong political support and constant pressure from the nation as a whole, which can be represented by local organizations or professional associations. However, the mere increase of available resources devoted to the health sector does not guarantee significant improvements of health outcomes of a population.

## 1. Introduction

Since 2000, the World Health Organization (WHO) has reported on the urgency of ensuring all populations proper health insurance.[1] In 2010, WHO released its report about health system financing and identified universal coverage as a critical element in attaining the right of every human being to enjoy "the highest attainable standard of health." Globally, many countries have struggled to ensure the well being of their citizens and to protect them from poverty. The countries that have come the closest to achieving universal coverage generally have more resources to spend on health. For example, a group of countries belonging to the Organization for Economic Co-operation and Development (OECD), had average annual per capita expenditures on health close to USD 4,000.[2] However, large expenditures on health is not required for a country to achieve universal coverage, for instance Thailand reduced the number of uninsured from 26.6% of the population in 2001 to 4.4% in 2005, though its health expenditure was below the average of richer countries who reached similar goals.[3] Thailand changed their method of collecting revenue from direct payment to pooling resources and used those funds to cover their entire population, especially the uninsured prior to the reform. Thus, reaching universal coverage for low-middle income countries is achievable, but it demands commitment and investments from the government and society.

Thailand is a developing country located in Southeast Asia, where most countries have struggled to escape the economic crisis from a decade ago. Like many developing countries, Thailand faced challenges of inequality in many areas, including providing universal access to health care. In early 2002, Thailand implemented universal coverage of health care for nearly all of their population by introducing "The Universal Coverage (UC) Scheme" funded by general tax revenues. The number of uninsured declined from 16.5 million in 2001 to 2.9 million in 2005.[3] Their success was based on the strong political commitment paired with scientific evidence by a group of researchers either commissioned by the government or worked independently as academia. Though they still face many challenges in terms of specific specialty health care services, Thailand has proven that developing countries have the ability to reform their health system to achieve "Health for All".

Meanwhile, Indonesia, another developing nation in Southeast Asia, has an entirely different situation. Since the economic crisis in 1998, Indonesia has implemented numerous reforms in regards to how health care should be delivered. A significant reform was the development of managed care schemes, called Jaminan Pemeliharaan Kesehatan Masyarakat (JPKM) in 1999. Unfortunately, poor outcomes and failures in the sustainability of the local health insurance scheme resulted in the premature termination of the reform.[4]

Now, private sectors still play a large role in delivering care and predominantly receive direct compensation from patients. In 2008, the Ministry of Health announced the new social health insurance program, called Jaminan Kesehatan Masyarakat (Jamkesmas), targeted at the poor.[5] Unfortunately, since 2001, the governance system that had been changed into decentralization has made the program implementation greatly varied, among the provinces.[6] Despite the significant strides over the past decade, Indonesia still has many challenges to reach its ultimate goal of universal health coverage.

In this paper, the author attempted to compare the health financing system between Thailand and Indonesia. Both nations are similar in regards to socioeconomic level. Both countries also suffered from the great financial crisis and International Monetary Fund bailout in 1997. Presently, both show significant economic improvement reflected in their GDP annual growth above 5%.[7] The objective of this paper is to examine each health system and identify lessons where economic power affects the health status of a given population.

## 2. Methodology

This descriptive study compares the health care system in both nations with special attention on how the countries finance their systems, and the health status of their people. Existing data were used to compare the health care systems, morbidity and mortality rates, and health workforce in each country. Relevant data were collected from online institutional databases. All statistical data were collected from various resources, such as World Bank, WHO and local health authority databases in each country. For analysis, the authors followed The Health Systems Assessment Approach: A How-To Manual Version 1.0, issued by Health Systems 20/20 and supported by the United States Agency for International Development (USAID).[8] The health finances of both countries were compared using specific assessment indicators provided in the framework.

The data were divided into four topical areas: 1.) Amount and sources of financial resources, 2.) Ministry of Health budget and expenditures, 3.) Health insurance and 4.) Out-of-pocket (OOP) payments. Due to the limited availability of the data, the authors only selected the first, third, and fourth points as the primary focus of this paper. Literature reviews were performed to discuss the results from the analysis. The authors used Google Scholar and PubMed as search engines and used several keywords: "Indonesia" or "Thailand" or "Health Financing". Information was gathered from various reports published by international organizations, such as the World Bank and WHO, and local organizations from both countries. Based on the findings, the authors provided a summary and developed recommendations, which may help to improve both health care systems.

## 3. Results

*Health Insurance*

Table 1 shows a comparison of the health insurance coverage between Thailand and Indonesia. In Thailand, there are three main categories of health insurance, which cover nearly all of the population: Civil servant benefit, formal worker insurance and universal coverage for the non-formal sector. The Civil Servant Medical Benefit Scheme (CVMBS) covers approximately nine percent of the population, consisting of government employees, retirees and their dependents. CVMBS is financed solely by general revenue taxes with no further contributions. Payment methods used for health care services are fee-for-service (FFS), with a reimbursement model for outpatient setting and Diagnosis Related Groups (DRG) for inpatient setting.[3,9]

**Table 1. Health Care Insurance Coverage in Indonesia's Population (2012)[3,17]**

| Health Insurance Type | Indonesia | | Thailand | |
|---|---|---|---|---|
| | Total* | Percentage | Total* | Percentage |
| Social Insurance for Civil Servant | 19.5 | 8.14% | 9.5 | 14.77% |
| Health Insurance Scheme for Poor Population | 108.2 | 45.14% | 45.3 | 70.45% |
| Labor Health Insurance | 5.6 | 2.34% | 5.9 | 9.18% |
| Private Health Insurance | 18.3 | 7.63% | 0.7 | 1.09% |
| Total Insured | 151.6 | 63.25% | 61.4 | 95.49% |
| Total Uninsured | 88.1 | 36.75% | 2.9 | 4.51% |
| Total Population | 239.7 | 100.00% | 64.3 | 100.00% |

*in millions

Approximately 15% of Thailand's population have formal jobs and are covered by two distinct types of insurance schemes: the Social Security Scheme (SSS) that covers non work-related diseases and the Worker Compensation Scheme (WCS) that covers only work-related diseases or injuries.[11] Both SSS and WCS are financed by a payroll tax with tripartite contributions (Employer, Employee, and Government) with 1.5% of base salaries from each. The SSS uses a capitation system and the WCS uses a fee-for-service reimbursement method for disbursing health costs to the providers. In addition, SSS does not have a referral mechanism for more specialized services and only covers the contributor, neither for their dependents.

In 2001, the Government launched Universal Coverage Scheme (UCS) that covered rest of the population (approximately 48.4 million people, or 76.6% of the population) who were ineligible for other public insurance schemes.[12] Originally, the UCS provided a comprehensive benefits package where all family members were eligible and covered both inpatient and outpatient services, with the exception of renal replacement therapy and anti-retroviral therapy for HIV/AIDS. However, due to strong social movement, the UCS was modified to include both treatments as of January 2008.[13] Beneficiaries are restricted to receive medical treatment with selected contracted providers, mostly public primary care units in the rural area (District Health System). The UCS is financed by general tax revenues, while payment methods are more complicated. Capitation methods are used for outpatient services and prevention services; Global budget and DRG are used for inpatient services. A yearly budget of 1,202 baht (USD 24.02) per person is allocated to hospitals and health centers to provide medical services in the coverage area. Each person pays 30 baht (USD 0.02) per service, but since 2006 the co-payment mechanism has been terminated.[14] The universal coverage scheme has successfully prevented beneficiaries from incurring liability for catastrophic health payments (4.7% in 2000 and 1.9% in 2006) and protects them from health-related financial burdens.[3] UCS combines capitation funding with a shift in delivery led by primary care to help keep costs under control. Health expenditure only contributes to 3.5% of GDP, relatively lower compared to the lower-middle income country group average of 5.6% of GDP.[15]

Private insurance is available for people who want additional medical benefits not covered by public insurance in which they are eligible to join. In 2001, approximately 1 million people in Thailand bought private insurance, but it declined to 0.7 million by 2005.[3] Since it is voluntary, beneficiaries are required to pay certain amount of premium before they can receive full-coverage from the insurers. Fee-for-service (FFS) is a commonly used payment method by insurers to providers for the accrued medical cost of their beneficiaries.

Indonesia has a long history of establishing public insurance programs with the intention of achieving universal coverage. Currently, there are three public health insurance schemes each targeting a different population: Askes (Asuransi Kesehatan/Health Insurance) for civil servants and their dependents, Jamsostek (Jaminan Sosial Tenaga Kerja/Social Security for Private Labor), and Jamkesmas (Jaminan Kesehatan Masyarakat/Community Health Insurance).[5] Indonesia attempted to establish another public health insurance adopted from the American Health Maintenance Organization (HMO) System, called JPKM (Jaminan Pemeliharaan Kesehatan Masyarakat/Community Health Maintenance Insurance). Unfortunately, this scheme faced critical problems during implementation due to its inability to sustain the necessary pooling in order to fund the scheme at the district and provincial level.[4,16]

Askes or Health Insurance for Civil Servants was the first established in 1968 under Presidential Instruction No. 230/1968 to finance and deliver health services to both active and pensioned civil servants, including their direct family members (spouse and up to the third child). In 2007, this state-owned enterprise covered 14 million people or approximately six percent of the total population.[5]

It requires members to contribute four percent of their base salary, in which the employee contributes two percent and the government contributes the remaining two percent on behalf of the employee. Askes provides comprehensive benefit package, including inpatient and outpatient services in addition to a packaged payment option for maternal health care. Beneficiaries are restricted to receiving medical treatment only from selected registered providers, mostly public hospitals and public health centers. Askes reimburses medical costs incurred by beneficiaries using a FFS scheme and negotiates prices with providers and pharmaceutical companies and issues a list of services and medications covered by Askes. As a consequence of Askes becoming the largest insurance company in Indonesia, it gains strong bargaining powers to reduce cost and invest unused premium costs in the program.

The second part of Indonesia's health insurance scheme is Jamsostek or Social Insurance for private workers. Essentially, its main purpose is to provide comprehensive health benefits to formal and informal employees and their dependents, although the coverage for the informal sector was not successfully implemented. The contribution is divided into two categories depending on marital status: singles must pay three percent of their base salary and members with dependents must pay six percent of their base salary with a limit of Rp 1 million (USD 110), regardless of status level. Beneficiaries can seek medical treatment from selected public and private providers. In 2009, Jamsostek covered 4.1 million people (only 2.4% of the total population).[5] This low coverage was mainly caused by the employer opt-out option for companies who desired better benefit plans than Jamsostek.[4] Due to its low coverage, Jamsostek was unable to expand their benefits leading to limited health services that could be offered. Though this plan claimed to offer a comprehensive health benefit package, it failed to cover many services, such as cancer treatment, heart surgery, renal dialysis, prosthesis, non-basic immunization, etc. Its inability to compete with many private insurances that have more comprehensive benefit packages with competitive premiums has made its progress in enrolling new beneficiaries in a very slow manner.

The third scheme and the only insurance program that covered more than 10% of the population is Jamkesmas or Community Health Insurance. It evolved from Askeskin, the predecessor of mandatory public health insurance scheme, in 2008 and was controlled by the Ministry of Health in the registration of the targeted population and management of the payment system. Now, Jamkesmas covers 76.4 million or 14.6% of the total population.[17] It is targeted to the poor and the identification of eligibility is based on individual and household financial capability. Jamkesmas is funded from general tax revenues with only Rp 6,000 per capita (USD 0.62). Though the scheme initially utilized a FFS reimbursement mechanism, Jamkesmas began transitioning to a DRG provider payment system in 2009. Its beneficiaries are able to seek care at public and private providers, though the covered ambulatory services are solely public. Surprisingly, it offers comprehensive benefit package including expensive medical treatments, such as renal dialysis and heart surgery and does not include a pre-existing condition policy. Researchers suggested that Jamkesmas made a significant impact reflected in increased total utilization of services by 50% for ambulatory care and about 106% for inpatient care.[18] Also, the rates of service utilization between the most affluent and the poorest populations have nearly equalized.

## Amount and Sources of Financial Resources

This section provides the results of descriptive analysis conducted on data collected from the World Development Indicators database in 1995-2010.[19]

Total Health Expenditure (THE) is the sum of all outlays for health maintenance, restoration or enhancement paid for in cash or supplied in kind. It consists of government and private sector expenditures on health. Both Thailand and Indonesia spent less than five percent of their GDP on health throughout a 16-year period. In spite of a small difference in their THE, Thailand has managed to cover nearly all of its population by implementing a universal coverage scheme in 2002. As seen in Figure 1, Thailand's THE increased significantly in 2002 as a result of the initial universal coverage scheme. In 2008, the THE increased again significantly as a consequence of a new policy that included renal treatment for end-stage kidney failure and HIV treatment, which were originally excluded due to high cost burden on the national budget.[11,13]

**Figure 1. Comparison between Public and Private Health Expenditures, as a Percentage of GDP**

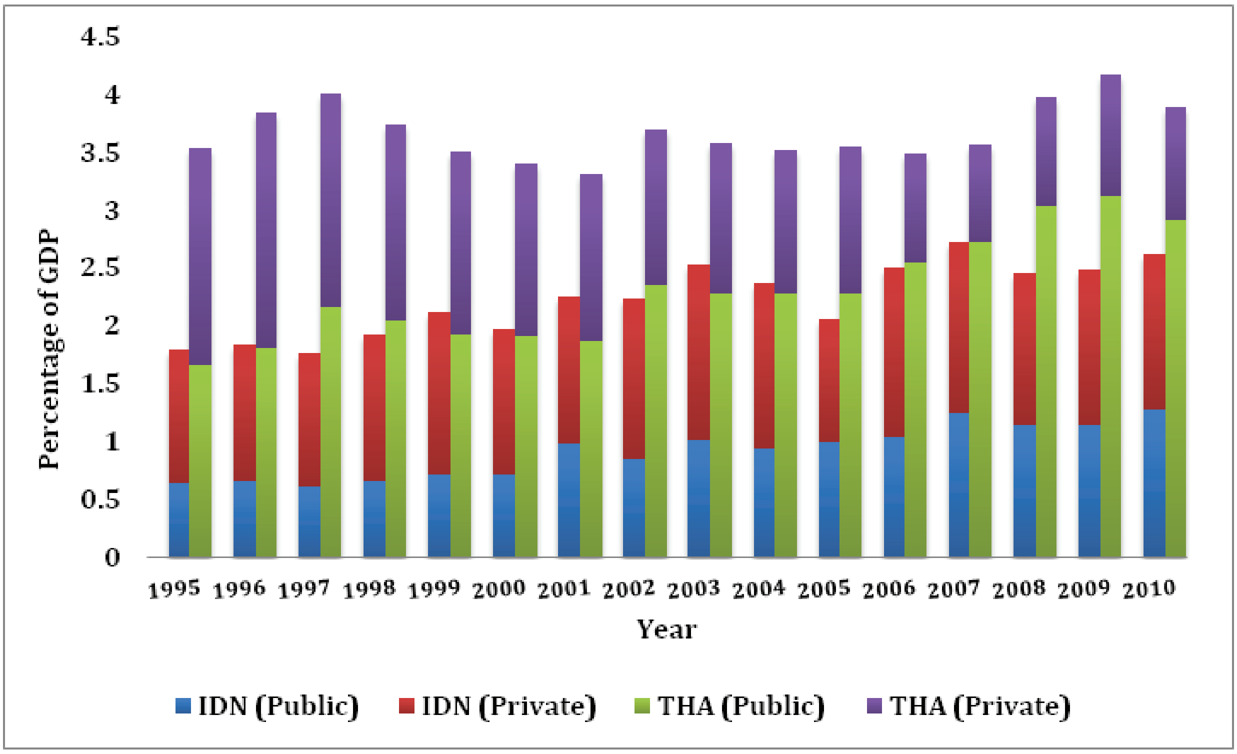

IDN = Indonesia; THA = Thailand; GDP = Gross Domestic Product

Note: The sum of public and private health expenditure represents the THE of respected country.

If we breakdown the total health expenditure into public and private sectors, we can observe that Thailand's government has played a dominant role in health expenditure, more than twofold that of the Indonesian government's share. Conversely, the Indonesian private sector contributed more to THE than its government, but the gap has reduced since the introduction of Jamkesmas in 2008. In 2010, both private and public health expenditure were nearly equal indicating that the Ministry of Health successfully balanced the role of government in providing health care.

Total health expenditure per capita expressed in purchasing power parity (PPP) terms or international dollars is an indicator that reflects the average amount of resources spent on health per person, measured in international USD. From Figure 2, we can observe a great difference between Indonesia and Thailand THE per capita.

**Figure 2. Health Expenditure per Capita as Purchasing Power Parity (PPP)**

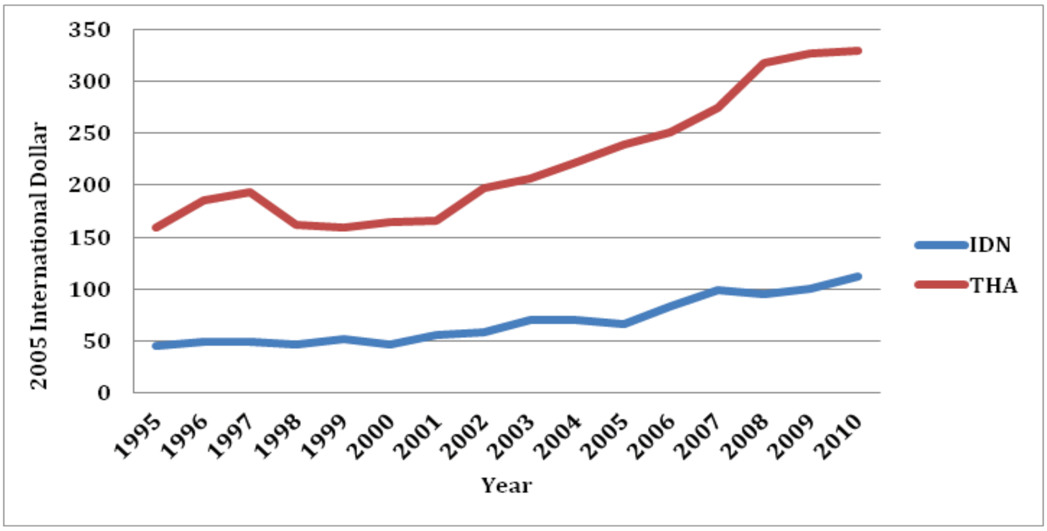

IDN = Indonesia; THA = Thailand; For PPP, the data used constant 2005 International Dollar

General government health expenditure (GGHE) is expressed as a percentage of total government expenditure. The Indonesian government committed less than 8% of the government budget on health. For comparison, since 2003 Thailand committed more than 10% of the government expenditure to invest in the health sector. Although both countries contribute less than 20% of their respective government expenditures on health, Thailand still has better health insurance coverage than Indonesia.

Another indicator shown in Figure 3 is public health expenditure as percentage of THE, which is a measure of the relative contribution of the central and local government relative to THE. From 1995 to 2010, Indonesia public shares on total health expenditure remained below 50%, in contrast to Thailand that spent more than 50% even before the implementation of universal coverage in 2002. It could mean that Indonesia has low tax capability to support the health sector or is heavily reliant on external funding, such as donor assistance in tuberculosis (TB) or Malaria programs.

**Figure 3. The Proportion of Public Health Expenditure in Indonesia and Thailand**

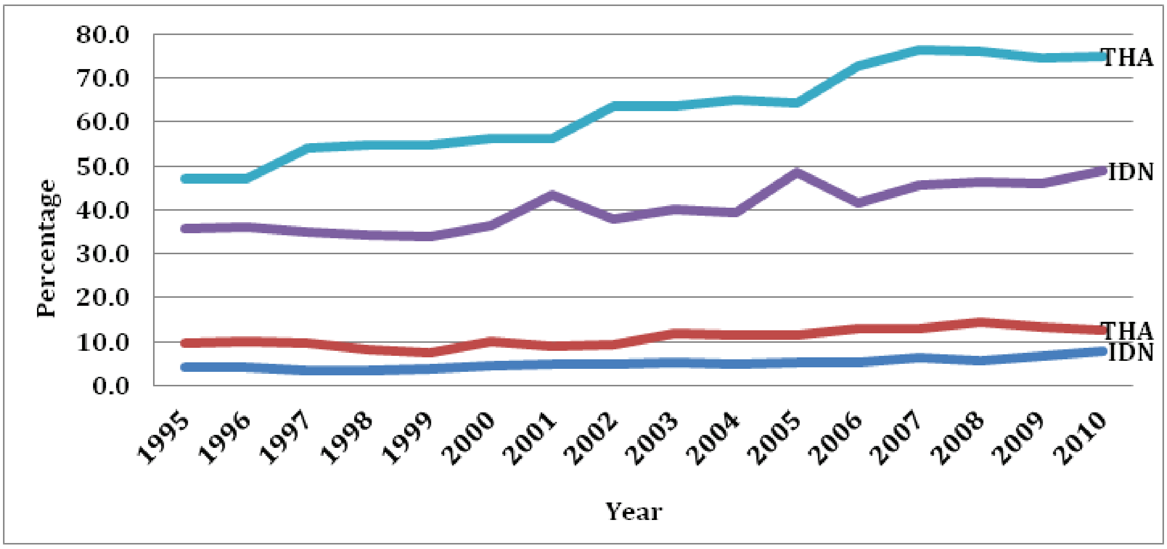

IDN = Indonesia; THA = Thailand
Note: The two bottom lines represent the public health expenditure as percentage of total government expenditure for each country. The two top lines represent the public health expenditure as a percentage of total health expenditure for each country.

The share of a country's THE financed by external sources measures the contribution of international agencies and foreign governments to THE. This indicator is shown in Figure 4. Thailand is less reliant on external sources in funding their health system. It is reflected in a trend of the percentage of external resources for health that has remained less than 0.5%. In contrast, external resources have a much larger role in funding Indonesia's health program, but it is not a major concern since this percentage has never reached 10%.

**Figure 4. External Resources for Health as a Percentage of THE**

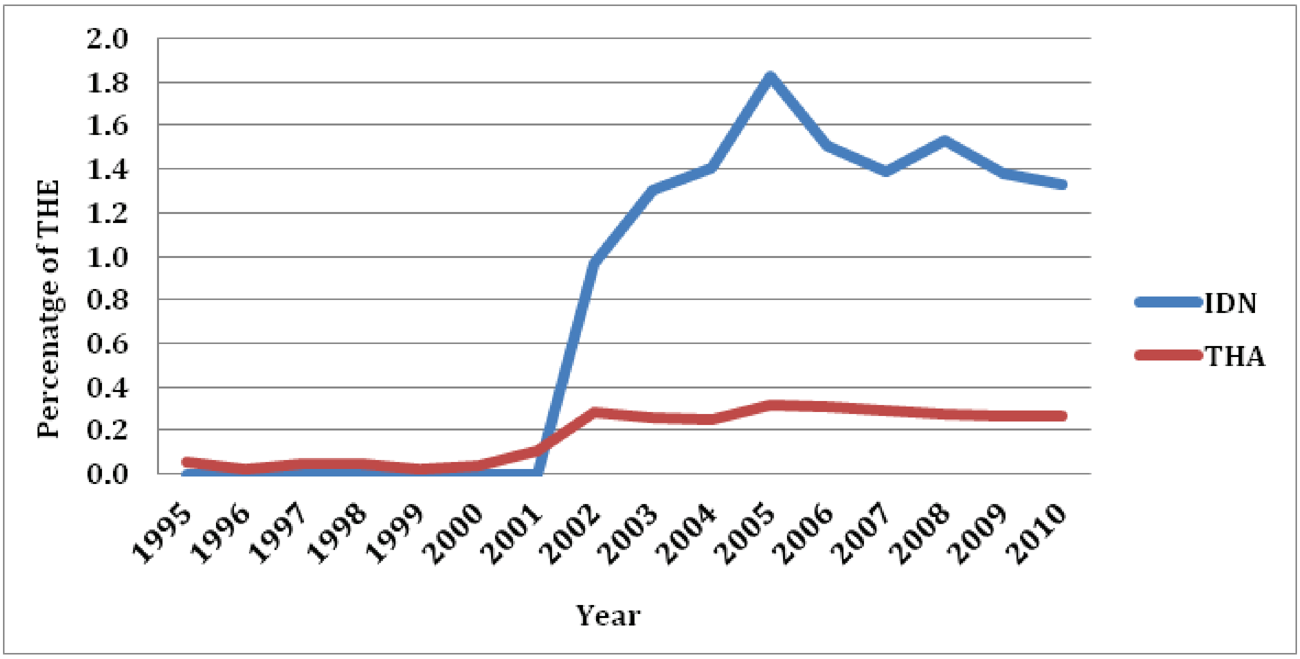

IDN = Indonesia; THA = Thailand; THE = Total Health Expenditure

OOP expenditure reflects household health expenditure and individuals health expenditure as a direct payment to health care providers irrespective of the sector and excluded from health insurance. This indicator is shown in Figure 5. When a country has a high percentage in this indicator, it shows that the country or sub-population of that country is more likely to incur catastrophic levels of health expenditure. Thailand has done a very good job in lowering the share of OOP in total health expenditure, as reflected in the declining trend in OOP percentages from 34% in 1999 to 14% in 2010. Indonesia's progress, on the other hand, has been gradual despite of the implementation of Jamkesmas in 2008 as a safety net program for the poor.

*Health Outcome and Health Workforce Density*

From Table 2, we can observe that Thailand had better health indicators in almost every aspect, except for HIV prevalence. Interestingly, Indonesia and Thailand had nearly similar densities of health personnel; with the exclusion of nurses and midwives, of which Indonesia had more. Despite the increased number of physicians and the improved ratio of physicians to population, deployment practices and inequitable distribution of services remain serious concerns in Indonesia.[20]

**Figure 5. Out-of-pocket Health Expenditure as Percentage of THE**

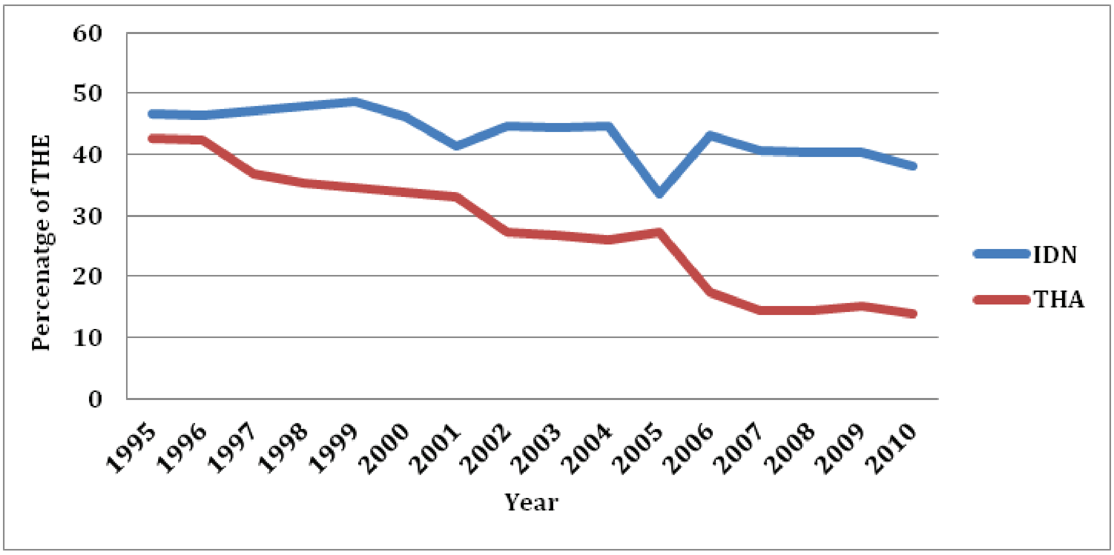

IDN = Indonesia; THA = Thailand; THE = Total Health Expenditure

**Table 2. Health Outcome and Health Workforce Density Comparison between Indonesia and Thailand[21]**

| Indicator | Indonesia | | Thailand | |
|---|---|---|---|---|
| | 1990 | 2011 | 1990 | 2011 |
| **Population Total** | 184,345,939 | 242,325,638 | 57,072,058 | 69,518,555 |
| **Age Distribution** | | | | |
| 0 - 14 | 36.46 | 26.73 | 30.09 | 20.18 |
| 15-64 | 59.78 | 67.64 | 65.28 | 70.73 |
| ≥ 65 | 3.76 | 5.63 | 4.63 | 9.09 |
| **Gender Distribution** | | | | |
| Male | 50.01 | 49.86 | 49.66 | 49.13 |
| Female | 49.99 | 50.14 | 50.34 | 50.87 |
| **Geographic Distribution** | | | | |
| Rural | 69.42 | 49.31 | 70.58 | 65.89 |
| Urban | 30.58 | 50.69 | 29.42 | 34.11 |
| **MDG-4 Indicator** | | | | |
| Infant Mortality Rate | 54 | 25 | 29 | 11 |
| Under Five Mortality Rate | 82 | 32 | 35 | 12 |
| Measles immunization coverage among 1-y.o (%) | 58 | 89 | 80 | 98 |
| **MDG-5 Indicator** | | | | |
| Maternal Mortality Rate | 600 | 220 | 54 | 48 |
| Birth attended by skilled health personnel (%) | 77.3 (2007) | | 99.4 (2009) | |
| Contraceptive Prevalence | 61.4 (2007) | | 81.1 (2006) | |
| **MDG-6 Indicator** | | | | |
| HIV prevalence among adults aged 15-49 years (%) | <0.1 | 0.2 (2009) | 1 | 1.3 (2009) |
| Antiretroviral therapy coverage among people with advanced HIV infection (%) | 24 (2010) | | 67 (2010) | |
| TB Incidence Rate | 187 (2011) | | 124 (2011) | |
| TB Prevalence Rate | 281 (2011) | | 161 (2011) | |
| TB mortality rate (excluding HIV) | 27 (2011) | | 14 (2011) | |
| **Health Workforce Density (per 10,000 population)** | | | | |
| Physician | 2.9 (2010) | | 3 (2010) | |
| Nursing and midwifery personnel | 20.4 (2010) | | 15.2 (2010) | |
| Dentistry personnel | 0.6 (2010) | | 0.7 (2010) | |
| **Hospital Bed Density (per 10,000 population)** | 6 (2010) | | 22 (2010) | |
| **Life Expectancy at birth (years)** | | | | |
| Male | 60 | 67 | 69 | 71 |
| Female | 64 | 71 | 76 | 77 |

MDG = Millennium Development Goals; HIV = Human Immunodeficiency Virus; TB = Tuberculosis

**4. Discussion**

One limitation of this study was the difficulty in data collection from earlier years due to limited access of national databases from both countries. The chosen framework required extensive data for developing an idea on how the Ministry of Health arranged the annual budget and how effectively the budget was spent each year. Nevertheless, this study can still provide key health financing indicators that are particularly useful in monitoring and tracking health-financing progress over time that can be compared between both countries.

In comparing the health financing of the two countries, we found that Thailand initiated its progress towards universal health coverage much earlier than Indonesia. Thailand had started to cover low-income populations since 1975 when the Low Income Card Scheme was launched. The most progressive move was in 2001 through the introduction of the "30 baht scheme" for universal access to subsidized health care services. Furthermore, in late 2002, The National Health Security Act was passed by Thailand parliament allowing for the civil service and social security for workers schemes to be merged with "30 baht scheme" into single universal coverage scheme.[9,12,14] Meanwhile, Indonesia had also enacted The National Health Security Act in 2004 that required the government to provide universal access to health care facilities.[22] Comparatively, each country enacted reforms moving toward universal health care relative to each other, still Thailand progressed further and achieved success in their health care reform much earlier than Indonesia, why? What did Thailand have prior to the health reform that Indonesia did not? Answering those questions is not easy since we need rigorous data to provide and support the actual answer. However, the author attempted to elaborate plausible explanations based on the data gathered in this study.

The first plausible explanation is the difference of political commitment by each government. The history of each country that successfully achieved universal coverage has similar patterns including widespread and persistent domestic pressures for the provision of universal coverage in Thailand. Universal coverage was one of the main political mandates under the leadership of Prime Minister Thaksin Chinawatra of the Thai Rak Thai (TRT) political party who won a substantial victory in the 2001 general elections.[12,23] The government successfully used the economic crisis as a turning point to reform their health care financing system. In addition, health bureaucrats led by reformists and researchers continuously supported the formulation and implementation of the reforms with rigorous evidence from domestic and international settings. The pressure from the bottom included health professional organizations, NGOs and university hospitals, who also took active roles in shaping the National Health Security Act into the legislation base of universal scheme.[14]

Meanwhile, Indonesia also took similar actions to reform its health care system by introducing a safety net program in 1999 after being hit by the economic crisis in 1997. The Ministry of Health also continued to expand and encourage the development of JPKM (similar to HMOs in the United States), which was based on community settings. Unfortunately, this program was evidently introduced prematurely and suffered from poor performance and lack of governmental support.[4,16] Individual institutional components were insufficiently developed for managed care to have a reasonable chance to succeed.[24] Consequently, key stakeholders became more reluctant on another attempt to reform health care services, as it was more difficult to regain their trust. This could be one reason why the movement towards universal coverage in Indonesia was slower than many of its neighboring countries. Even though the parliament had passed the National Health Security Act in 2004, its impact on preventing impoverishment due to high out-of-pocket payments was not seen until the introduction of Jamkesmas for the poor in 2008, which covered approximately 76.4 million population (31.9% of the total population). Nonetheless, the number of uninsured is still very high

(36.8%) and are at risk of impoverishment due to catastrophic health expenditures.[22]

The second reason for the slower progression of Indonesia in reforming its health care services is possibly due to the impact of decentralization on health authorities throughout the country. When the Indonesian Legislative passed the Regional Autonomy Act in 2002, many experts were concerned with the inequity in access to essential public health services, such as maternal and child health services, immunization, disease surveillance and control, including response to epidemics. When the decentralization was first introduced, policy makers in the central government expected the local government, especially the district who had larger fiscal capacity, to finance their own health expenditures more adequately and efficiently with little additional funding from the central government.[6,25] Unfortunately, the confusion between the decentralization of power and fiscal responsibilities made local funding varied and unequal among the local government. The interesting fact was that the highest budget for health among the local districts did not come from the richest district, but rather from the district where the governor envisioned a greater need and awareness of health for their people.[25] Meanwhile, Thailand also implemented a decentralization policy in 1999 with the issuance of The Decentralization Act. It mandated that all public services, including health services, should be the responsibility of Local Governments (LG). The country's approach to decentralization is considered delayed since there has been limited progress especially in health care decentralization during the past decade. As a result of delayed decentralization, recently some LGs with sufficient financial availability started to build their own health facilities and recruited health staff for their operation. These infrastructures were duplicative alongside existing health care infrastructures of the Ministry of Public Health (MoPH), which should have been devolved into LGs to increase the overall efficiency by reducing the bureaucratic costs.[26]

As mentioned, Indonesia, while on the right path, moved at a slower pace than Thailand. The introduction of Jamkesmas in 2008 brought hope to the poor who could not afford expensive medical treatments such as chemotherapy for cancer or emergency obstetrics for antenatal hemorrhages. Despite this massive effort, the number of uninsured remained high, at approximately 36.8% of the total population. Most of the uninsured were ineligible for Jamkesmas and Askes, and are not enrolled in private insurance due to expensive premium or lack of information about health insurance. Moreover, the number of self-employed people who worked either in rural areas (farmers, fisherman) or urban areas (retailer, vendor, taxi/motorcycle driver, etc.) and their family are usually at risk of suffering from catastrophic health expenditures because they often seek medical help only when diseases have reached later stages requiring more expensive medical treatments. The exact number is hard to predict due to poor data management in health care industries, but it is important to note that without a proper health benefit scheme administered by government, it is unlikely that the masses will enroll in private health insurance to protecting them from impoverishment due to catastrophic health expenditures.[27,28]

In regards to THE as a percentage of GDP, Indonesia continues to utilize less than 3% of GDP, in contrast with Thailand who, since 1995, continues to utilize more than 3%. Although the difference in THE as a percentage of GDP between these two countries was not very large, Thailand has better coverage for their health insurance system. This may indicate better management by health authorities or utilization in Thailand in covering targeted population and administering health services. Unfortunately, the data presented in this paper is not sufficient to infer that health care reform in Thailand is due to better performance as compared to Indonesia.

Conjunctively, Thailand showed a significant declining trend in the proportion of out-of-pocket health expenditures as a percent of total expenditure on health, especially since the universal coverage scheme took place in 2002. Meanwhile, Indonesia also showed declining trends, but at a much slower pace than

Thailand, even after the introduction of Jamkesmas in 2008. It indicated that the utilization of the scheme by the targeted population was still low. Possible explanations include that not all of the targeted population received the membership or the poor quality of public health center forced the poor to seek help in private practices not covered by the scheme. Surprisingly, data from the Ministry of Health regarding the utilization of the Jamkesmas budget from central to local government in 2012, revealed that out of 2.49 trillion Rupiah (approx. USD 258 million) budgeted to 497 health districts, of which only 366 districts had given the report by November 2012, they only used 883 billion rupiah (approx. USD 91.2 million), while more than half of the utilized funds were used for maternal health scheme (approx. USD 67.5 million).[29] It showed that local government could not absorb the allocated budget efficiently; furthermore, the quality of the health care services remained low.

The quality of health care services, especially in the public setting posed other problems. Based on author experience, the difference in the quality of services between public health centers and private practices is quite different and can potentially force the poor to seek treatment at private facilities. The way the system was designed created challenges for public health centers in providing the best services due to many internal factors. First, primary health centers (PHCs) suffered from shortages of general practitioners who were eager to stay in PHCs for longer term, especially in rural areas. The problem became more complicated with the abolishment of the regulation that mandated all newly trained medical doctors to spend 1 to 3 years of service in rural areas. Data from the Ministry of Health revealed that out of 9,315 health centers, 1,193 centers did not have a doctor (12.81%), with significantly worse conditions in East Indonesia (62.35-88.10%). Nurses and midwives were in better condition (3.02% and 3.95%), but dentists were the worst (46.69%).[30] The condition of PHCs in urban areas was not entirely better than that of rural areas. The ratio of general physicians was very small, forcing them to reduce the time of visits per patient to approximately 1-2 minutes. It created a stigma that health care services in PHCs were only for very poor people. If the public loses their trust toward public health centers, it creates a greater challenge for the government to introduce universal access for all, which required using public facilities rather than private ones. Additional evaluation is necessary to justify this finding, and to seek alternative ways to counter this problem.

If we observe the graphics comparing public and private health expenditure as a percentage of GDP, Thailand obviously had a very high public proportion compared to private, especially since 2002 when the "30 baht for all" was first introduced. In contrast, Indonesia always had a higher proportion in the private sector compared to the public. Fortunately, the two graphs recently became increasingly similar due to the introduction of Jamkesmas for the poor. Though Thailand just began its universal coverage scheme in 2002, the public sector had already shown high numbers since 1995. It may be an effect of the previously introduced Low-Income Card scheme in 1975, Voluntary Health Card scheme in 1985, or the expanded coverage of Low-Income Card scheme in 1992.

When examining the level of government health expenditure, expressed as a percentage of total government expenditure and a percentage of THE, Thailand and Indonesia showed increasing trends over time. However, since 2008, heatlh care expendiures in Thailand showed a significant decreasing pattern until 2010, in contrast to Indonesia, which showed a significant increasing pattern since 2008. It was unusual as Thailand had decided to include end-stage renal replacement therapy, a high-cost medical treatment, into the universal coverage scheme since 2008.[13] A possible explanation is that Thailand was attempting to contain the cost of administering the universal coverage scheme. Further study is needed to justify this finding.

For the past 10 years, especially after the economic crisis in 1997, Indonesia received a substantial amount of funding from an external donor. The funding was targeted to be used toward specialized diseases or the health sector, in areas such as TB Malaria, HIV/AIDS, and maternal and child health. The Millennium Development Goals attracted many donors lending their help to Indonesia. However, the percentage of external resources in the total expenditure on health was still in the acceptable range. It meant that the government still had the financial capacity to provide health care services from domestic resources. Thailand obviously showed very little dependence on external funding, reflected by its low percentage (always below 0.5%).

## 5. Conclusions

In this study, it was evident that Indonesia can learn from Thailand on how to initiate, formulate, implement and evaluate policies to provide universal access of health care services. One important lesson is that health care reform is difficult to achieve without strong political support and constant pressure from society, which can be represented from a local organization or professional association.[31] Hopefully, the progress of achieving universal health care in Indonesia will be faster in 2014 when the Askes, Jamsostek and Jamkesmas will dissolve into one board called the BPJS (Social Security Administrator Board).[17] However, the mere presence of higher budget allocation itself cannot guarantee significant improvement in health care outcomes for an entire population. Further study is critical to evaluate the sustainability of health care reform policies in the future and predict the next step needed to counter any problems related to this reform.

## Conflict of Interest Declaration

The authors have no conflicts of interest to declare.
